# Effect of bio‐fertilization and edapho‐climatic conditions on chickpea nodulation, grain yields, and seed protein in a Mediterranean context: a meta‐analysis approach

**DOI:** 10.1002/jsfa.70430

**Published:** 2026-01-07

**Authors:** Amira Hachana, Amir Souissi, Lewis Ziska, Katherine Morgan, Carina Yiu, Issam Achour, Asma Bouatrous, Imen Hemissi, Hanen Arfaoui, Anissa Riahi, Manel Bouraoui, Bouaziz Sifi

**Affiliations:** ^1^ National Institute of Agricultural Research of Tunisia, Laboratory of Agricultural Sciences and Techniques Carthage University Tunis Tunisia; ^2^ Swift Current Research and Development Centre Agriculture and Agri‐Food Canada Saskatchewan Canada; ^3^ Department of Environmental Health Sciences Columbia Mailman School of Public Health New York NY USA; ^4^ National Agronomic Institute of Tunisia, Department of Rural Engineering, Water and Forestry Carthage University Tunis Tunisia; ^5^ Higher Institute of Biotechnology of Beja, University of Jendouba Beja Tunisia; ^6^ Higher School of Agriculture of Kef, University of Jendouba Jendouba Tunisia; ^7^ National Institute of Agricultural Research of Tunisia, Horticulture Laboratory Carthage University Tunis Tunisia

**Keywords:** chickpea, edapho‐climatic variations, grain yield, nodulation, rhizobial inoculation, seed protein

## Abstract

Chickpeas (*Cicer arietinum* L.) serve as a protein‐rich staple, particularly in Mediterranean countries, where they are often grown in marginal and water‐stressed areas. This meta‐analysis synthesized evidence from peer‐reviewed publications across Mediterranean countries to assess how chickpea rhizobial inoculation influences chickpea performance under different edapho‐climatic conditions. The results demonstrated that the highest impact of rhizobial inoculation on nodule number (NN) occurred under semi‐arid conditions (+34.98 nodules). Under sub‐humid conditions, the most substantial gains of inoculation were observed in nodule dry weight (NDW) (+135.44 mg plant^−1^), shoot dry weight (SDW) (+7.67 g plant^−1^), and grain yield (GY) (+906 kg ha^−1^). Regarding soil types, fine‐textured soils showed the most significant inoculation benefits for NN (+36.54 nodules) and NDW (+212.63 mg plant^−1^), whereas medium‐textured soils showed the highest increase in SDW (+9.21 g plant^−1^) and GY (+1517.02 kg ha^−1^). Additionally, alkaline‐pH soils demonstrated the highest inoculation impact on SDW (+7.65 g plant^−1^) and GY (+1054.78 kg ha^−1^). While soil salinity had a significant beneficial effect only on SDW in no‐saline soils (+4.36 g plant^−1^), soil phosphorus (P_2_O_5_) showed a positive impact on both SDW (+7.59 g plant^−1^) and GY (+1527.6 kg ha^−1^). For seed protein content (SP), rhizobial inoculation showed no significant effect. However, co‐inoculation of *Mesorhizobium* sp. and *Enterobacter* sp. consortium showed a promising improvement in NN, GY, and SP. Rhizobial inoculation showed a promising effect on Mediterranean chickpea performance. However, to address edapho‐climatic challenges while ensuring potential contribution to soil health and food security, further research on tailored and adapted inoculants is mandatory. © 2026 The Author(s). *Journal of the Science of Food and Agriculture* published by John Wiley & Sons Ltd on behalf of Society of Chemical Industry.

## INTRODUCTION

Climate change in the Mediterranean region is exacerbating drought, salinity, and floods, as a result of rising temperatures, potential evapo‐transpiration, and greenhouse gas emissions. The effects of the climate crisis give rise to soil degradation, biodiversity losses, and global effects on agricultural production.^1^, ^2^, ^3^, ^4^ Furthermore, conventional farming practices, particularly the intensive use of N‐P‐K fertilizers and pesticides, contribute significantly to the environmental crisis by affecting water and soil quality, as well as increasing CO_2_ emissions.^5^, ^6^ To mitigate these negative impacts, adopting sustainable and climate‐resilient agricultural practices is crucial.^7^ Leguminous crops, such as chickpea (*Cicer arietinum* L.), are a paramount component of sustainable agriculture due to their unique ability to provide multiple ecosystem services.^2^ Chickpea is widely cultivated around the world, particularly in temperate, arid, semi‐arid, and subtropical environments.^8^, ^9^, ^10^ Its cultivation is documented to improve soil fertility and health due to chickpea's ability to fix atmospheric nitrogen (N_2_), meeting up to 87% of its total N requirements, demonstrating the potential to optimize sustainable nutrient cycling.^8^, ^11^, ^12^

Chickpea association with beneficial soil bacteria of the genus *Rhizobium*, such as *Mesorhizobium cicer* and *Mesorhizobium mediterranum*, can fix 4–141 kg ha^−1^ N, depending on environmental conditions.^8^, ^11^ Additionally, chickpea inoculation with *Mesorhizobium* sp. strain showed increased plant dry matter, nodule number, seed yield, and grain protein.^13^, ^14^ Moreover, the synergic effect of rhizobia with other free‐living bacteria, identified as plant growth‐promoting bacteria (PGPB), such as *Bacillus* sp., *Pseudomonas* sp., *Enterobacter* sp., and *Burkholderia* sp., has demonstrated a positive effect on chickpea plants' growth and nitrogen accumulation, flower, pod, and seed production, as well as seed protein content, increasing their resilience against biotic and abiotic stresses.^15^, ^16^, ^17^, ^18^, ^19^, ^20^ Furthermore, dual inoculation of *Mesorhizobium* sp. with PGPB has been found to be more effective on chickpea and other crops than single inoculation, due to the synergetic effect of providing a more balanced nutrition and better adaptation to biotic and abiotic stresses.^16^, ^20^, ^21^, ^22^, ^23^ These PGPB are implicated as important biogeochemical processes in the agroecosystems through enhancing nitrogen fixation, phosphate and micro‐nutrient solubilization, phytohormone production, plant defense and nutrient acquisition, and improving soil fertility, resulting in improved plant growth and yield.^5^, ^13^, ^21^, ^24^

In the Mediterranean basin, chickpea is often cultivated in marginal lands that may be low in native specific rhizobia.^11^, ^12^, ^25^ It ranks as the second most cultivated grain legume after faba beans, covering 617.8 thousand hectares, with a total production of 685.4 thousand tons and an average productivity of 1167.7 kg ha^−1^ in 2022. The ten‐year mean chickpea production in main Mediterranean countries includes Turkey (528.6 tons), Morocco (48.7 tons), Syria (41 tons), Algeria (34 tons), Italy (19.6 tons), Greece (11.2 tons), Tunisia (7.8 tons), and Egypt (3070 tons).^26^, ^27^ In these countries, the common ‘Kabuli’ cultivar is predominantly cultivated under rainfed conditions in rotation with wheat, maize, oat, rice, sorghum, etc.^9^, ^28^, ^29^ Chickpeas are sown from February to mid‐April for spring cultivars, but can be planted as early as January for certain newly developed winter cultivars. These cultivars are challenged by yield instability due to abiotic and biotic stresses like terminal drought, cold stress, fungal diseases (e.g., *Fusarium* wilt and *Ascochyta* blight), pests, and parasitic plants (e.g., *Orobanche crenata* and *Orobanche foetida*).^22^, ^30^, ^31^, ^32^

Additionally, chickpea yields are adversely affected by climate change variability across Mediterranean agroecological environments, and variation in rainfall and temperature patterns.^33^ Such environments negatively impact rhizobia–legume symbiosis efficiency, leading to impaired nodulation performance, low symbiotic nitrogen fixation, and, consequently, low grain yield and quality.^34^, ^35^ Further, soil degradation, low water and nutrient availability, salinity, soil acidity and alkalinity, as well as extreme temperature, are detrimental for microbial activity including *Rhizobium*.^23^ Therefore, the isolation of native rhizobia, assessment of their efficiency through inoculation, and study of the effect of environmental factors on chickpea production under Mediterranean conditions were the topic of several research investigations, showing significantly higher above‐ground dry matter and yields produced in inoculated chickpeas than the uninoculated controls.^34^ These studies revealed an improvement in native rhizobia‐inoculated chickpea performance, attributed to the increased supply of N through enhanced biological N fixation compared to non‐inoculated plants. Meanwhile, N_2_ fixation efficiency showed different patterns due to several on‐farm determinant factors for rhizobial inoculation efficacy. They include environmental factors – mainly soil pH, N–P availability, native rhizobia, and climate (water stress, high temperature) – formulation factors, strains' effectiveness and inoculum quality (concentration and age), host factors – namely symbiotic effectiveness and stress tolerance – as well as agronomic factors, including sowing date, pest control, and tillage practices.^36^

The current meta‐analysis synthesized evidence from 27 peer‐reviewed publications across ten Mediterranean countries to assess how rhizobial inoculation of chickpeas, relative to non‐inoculation, influences nodulation, plant biomass, grain yield, and seed protein concentration under different edaphic and climatic conditions; and to analyze the effects of different inoculum types on chickpea nodulation, growth, and yield performance in order to identify future research perspectives needed to optimize inoculation efficiency under various agroecological conditions in the Mediterranean region.

## MATERIALS AND METHODS

### Data collection and extraction

This meta‐analysis fills a data gap on the effect of chickpea inoculation with rhizobia and other plant growth‐promoting rhizobacteria (PGPR) on nodulation, growth, grain yield, and seed protein under Mediterranean conditions. The bibliographic search was conducted in the Web of Science database (WOS, https://www.webofscience.com/wos/zoorec/basic-search), addressing relevant peer‐reviewed articles from 1990 to 2024. We have chosen to rely exclusively on the WOS database due to its extensive multidisciplinary coverage, rigorous journal selection criteria, and relevance to agricultural and environmental sciences, providing representative literature sufficient for valid meta‐analysis. The bibliographic search was conducted up to January 1, 2025. The following Boolean search of key terms and their combinations was used to capture relevant articles: (‘chickpea’ OR ‘C*icer arietinum’*) AND (‘rhizobia’ OR ‘PGPR’ OR ‘inoculation’, (‘nodulation’ OR ‘yield’ OR ‘protein’) AND (‘Mediterranean’ OR ‘Algeria’ OR ‘Croatia’ OR ‘Egypt’ OR ‘Greece’, ‘Italy’ OR ‘France’ OR ‘Lebanon’ OR ‘Morocco’ OR ‘Libya’ OR ‘Portugal’ OR ‘Spain’ OR ‘Syria’ OR ‘Tunisia’ OR ‘Turkey’).

From an initial pool of 100 articles, a total of 27 papers met the selected inclusion after screening titles, abstracts, and carefully examining experimental trials (Table 1). From these 27 included publications, 821 lines or observations corresponding to stratified data with targeted criteria were grouped into field experiments conducted under rain‐fed and conventional tillage systems. Studies were included in the meta‐analysis only if they met all of the following criteria: (i) open field cropping data; (ii) rain‐fed experiments; (iii) conventionally conducted trials; (iv) the experimental treatments consisting of rhizobial inoculation (*Mesorhizobium* sp. or *Rhizobium* sp.), rhizobial inoculation combined with PGPR (*Bacillus* sp. or *Enterobacter* sp.), PGPR inoculation lone, and non‐inoculated treatment (control); (v) measurement and reporting of at least one of the following variables : number of nodules, nodules dry weight, shoot dry weight, grain yield, or seed protein in inoculated or non‐inoculated data. The locations of the sampling sites are mapped in Fig. 1. Preliminary analysis of the selected database revealed that the peer‐reviewed articles were unevenly distributed among the Mediterranean countries: Algeria (1 article; 12 lines), Croatia (1 article; 42 lines), Egypt (1 article; 133 lines), Greece (2 articles; 25 lines), Italy (0 articles), France (0 articles), Lebanon (0 articles), Morocco (04 articles; 112 lines), Libya (0 articles), Portugal (01 article; 65 lines), Spain (04 articles; 74 lines), Syria (01 article, 15 lines), Tunisia (09 articles; 216 lines), and Turkey (5 articles;126 lines). These insights provide an overview of research progression on our specific topic and underscore the scarcity of impacted bibliographic resources in the Mediterranean region.

**Table 1:** List of studies used in the meta‐analysis

Reference	Country	Observation number	Climate	Mean temperature (°C)	Rainfall (mm)	Soil texture	Inoculated (bacteria genera)/non‐inoculated
Kherif *et al*. (2023)^37^	Algeria	12	T	14.27; 13.15; 12.72; 16.64	257.22; 259.55; 272.25; 274.2	Clay loam	Non‐inoculated
Vollheyde *et al*. (2024)^38^	Croatie; Spain; Tunisia	91	T; SA	18; 18.1; 18.4; 20.32; 20.81	97; 99.5; 125.6; 188.5; 225.6; 352.8	—	Inoculated (*Rhizobium* sp.); non‐inoculated
Soliman *et al*. (2012)^39^	Egypt	133	SA	—	—	Sandy clay loam	Non‐inoculated
Koutroubas *et al*. (2009)^40^	Greece	17	T	16.8; 17.69	189.1; 210	Silty clay	Non‐inoculated
Koutroubas *et al*. (2024)^41^	Greece	8	T	18.73; 18.98	196.6; 483	Silty clay	Non‐inoculated
Benjelloun *et al*. (2021)^42^	Morocco	32	SA	17.1	449	Clay	Inoculated (*Mesorhizobium* sp.; *Bacillus* sp. *Enterobacter* sp.; *Mesorhizobium* sp. + *Bacillus* sp.; *Mesorhizobium* sp. + *Enterobacter* sp.); Non‐inoculated
Devkota *et al*. (2021)^43^	Morocco	42	T	15.8; 15.4; 13.3	214; 276; 509	Clay loam	Non‐inoculated
Khadraji *et al*. (2020)^44^	Morocco	10	T	—	—	—	Non‐inoculated
Sadiki and Rabih (2001)^45^	Morocco	28	SA	—	—	Clay loam; Silty clay	Non‐inoculated
Duarte (2022)^46^	Portugal	65	T	—	From 130.75 to 824.6	—	Non‐inoculated
Lopez‐Bellido *et al*. (2004a)^47^	Spain	9	T	18.4: 20.35; 18.55; 20.9	207.407; 181.481; 229.63; 74.074	Clay	Non‐inoculated
Lopez‐Bellido *et al*. (2004)^48^	Spain	21	T	18.85; 17.25; 18.925; 19.55; 18.55; 20.9	100; 92.6; 51.9; 230; 144; 207; 181; 40.7; 81.5; 133; 74.1; 104	Clay	Non‐inoculated
Valverde *et al*. (2006)^49^	Spain	4	T	—			Inoculated (*Mesorhizobium* sp.); Non‐inoculated
Oweis *et al*. (2004)^50^	Syria	15	T	17.08; 12.1; 9.94; 11.85	408; 305; 222; 383	Clay	Non‐inoculated
Ben Mbarek *et al*. (2009)^51^	Tunisia	45	SA	22.4	46.7	Sandy loam	Non‐inoculated
Ben Romdhane *et al*. (2007)^52^	Tunisia	60	SH; SA	—	—	Clay; Sandy Clay loam	Inoculated (*Mesorhizobium* sp.); Non‐inoculated
Ben Romdhane *et al*. (2008)^53^	Tunisia	64	SA; A	—	—	Clay; Sandy Clay loam	Inoculated (*Mesorhizobium* sp.); non‐inoculated
Ben Romdhane *et al*. (2009)^54^	Tunisia	2	T		—	Clay	Inoculated (*Mesorhizobium* sp.); Non‐inoculated
Ben Romdhane *et al*. (2022)^55^	Tunisia	4	SA	—	—	Clay	Inoculated (*Mesorhizobium* sp.); Non‐inoculated
Hemissi *et al*. (2015)^56^	Tunisia	12	SH	19	361.6; 556.2	Clay; Clay loam	Inoculated (*Mesorhizobium* sp.); Non‐inoculated
L'taeif *et al*. (2009)^57^	Tunisia	16	SH	19	550.9. 269. 463.3. 256.4	Clay; Clay loam	Inoculated (*Rhizobium* sp.); non‐inoculated
Ouji *et al*. (2016)^32^	Tunisia	4	SA; T	14.8	286.4	Sandy clay loam	Non‐inoculated
Güngör (2024)^58^	Turkey	70	T	9.3; 10.65; 12	—	—	Non‐inoculated
Kantar *et al*. (2003)^59^	Turkey	14	H	30.3; 19.3; 19.8	125.2; 60.4; 116.6	Sandy loam	Inoculated (*Rhizobium* sp.); non‐inoculated
Karasu *et al*. (2009)^60^	Turkey	12	T	17.95	175.5; 279.2	Clay loam	Inoculated (*Rhizobium* sp.); non‐inoculated
Kayan *et al* (2017)^61^	Turkey	18	H	12.65; 11.13	338.5; 546.1	Loam	Non‐inoculated
Yagmur and Kaydan (2011)^62^	Turkey	12	T	—	98.4; 63.7	Sandy clay loam	Inoculated (*Rhizobium* sp.); non‐inoculated

A, arid; H, humid; SA, semi‐arid; SH, sub‐humid; T, temperate.

**Figure 1:**
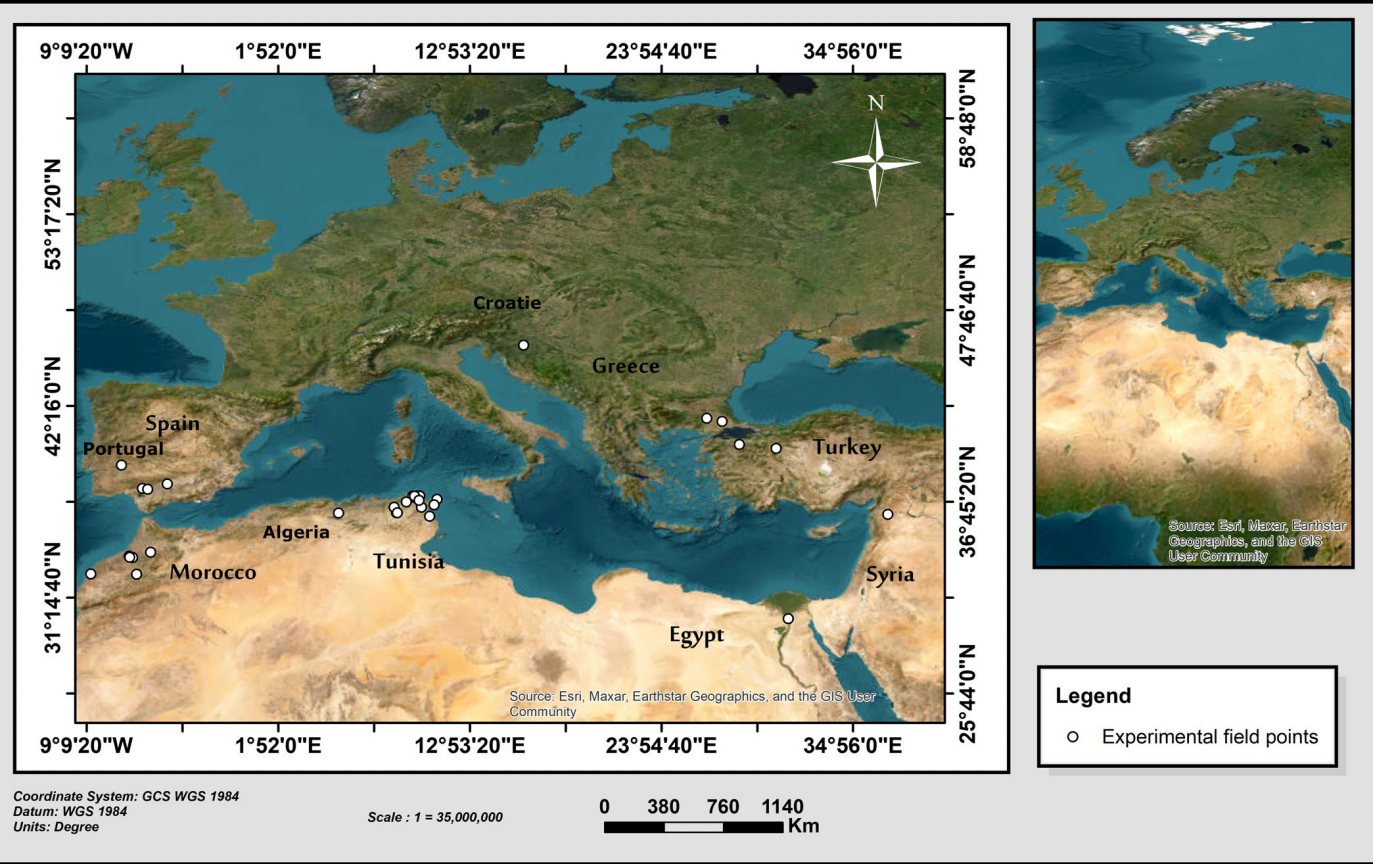
Geographical locations of the sampling sites used in the meta‐analysis.

### Data grouping

The studies were classified into five bioclimatic zones: humid (H), sub‐humid (SH), semi‐arid (SA), arid (A) and temperate (T). The data are stratified by bioclimatic zone, soil texture, soil pH, electrical conductivity, assimilable phosphorus, experimental treatment (inoculated/non‐inoculated), and inoculum type. The soil textures were classified into three groups of fine (clay and clay loam), medium (silty loam and sandy clay loam), and coarse (sandy loam).^63^, ^64^, ^65^ The pH was grouped into three categories of acid (pH < 6.5), neutral (6.6 < pH < 7.3), and alkaline (pH > 7.3) soil pH. The electrical conductivity was grouped into two levels of non‐saline (EC < 0.2 S m^−1^) and slightly saline (0.2 S m^−1^ < EC < 4.5 S m^−1^) soil. The soil phosphorus richness was grouped into two levels of low (P_2_O_5_ < 43 mg kg^−1^) and medium (44 mg k g^−1^ < P_2_O_5_  65 mg k g^−1^) soil phosphorus richness.

The targeted variables extracted from the studies are nodule number (NN), nodule dry weight (NDW), shoot dry weight at flowering (SDW), grain yield (GY), and seed protein content (SP). For each study, information about author, country, site geographical location, climatic parameters (bioclimate, rainfall, temperature, evapotranspiration), pedological parameters (texture, clay, silt, sand, site, total nitrogen, available P, exchangeable K, electrical conductivity, pH, active limestone, and organic matter), cultivar, sowing rate and season, NPK fertilization rates, and inoculated bacteria species were collected.

### Meta‐analysis

We calculated the mean difference (MD, Eqn (1)) of NN, NDW, SDW, GY, and SP was calculated to compare the inoculation at different variations of edapho‐climatic conditions (climate, soil texture, pH, EC, and P_2_O_5_).

MD is a statistical parameter that measures the absolute difference between the mean value of two groups. It estimates the amount by which an experimental treatment modifies the result, on average, compared to another treatment. The data required for estimation of the MD are in the form of treatment mean (*μ*), standard deviation (*σ*), and number of repetitions (*n*).

We determined edapho‐climatic factors as key moderating variables that might influence inoculation effectiveness. Therefore, we analyzed how these factors affected the magnitude of the observed mean differences across studies (Eqn (1)):

(1) $$\mathrm{MD}=\mu_{i1}–\mu_{i2}$$

where *µi*_1_ and *µi*_2_ are noculated and non‐inoculated trials, respectively.

A random effect model analysis was performed with R version 4.1.2,^66^ using the ‘meta’ package^67^, ^68^ to calculate the MD and represent the results *via* forest plots.

To compare the studied parameters at different inoculum types, random‐effects meta‐analysis was performed using the ‘metafor’ package^69^ in R (version 4.1.2), employing the restricted maximum likelihood (REML) method to estimate heterogeneity, which produces unbiased estimates of variance components in random‐effects models by limiting the likelihood to the variance parameters while eliminating fixed effects.

## RESULTS

### Effect of rhizobial inoculation on chickpea nodulation, growth, and yield parameters

#### Effect of rhizobial inoculation on chickpea NN and SDW

Overall, rhizobial inoculation revealed a positive effect on chickpea nodulation and plant growth. This is evidenced by an increase in NN and SDW of 218.2% and 38.88%, respectively, relative to non‐inoculated controls. The climate revealed a significant effect on NN response to rhizobial inoculation (*P* < 0.01; Fig. 2). The highest positive response was observed under semi‐arid climate (MD = 34.98 nodules plant^−1^), followed by a moderate response under sub‐humid and arid climates (MD = 22.47 and 18.25 nodules plant^−1^, respectively), and the lowest under temperate climate (MD = 13.38 nodules plant^−1^). Soil texture significantly influenced NN response to rhizobial inoculation (*P* < 0.0001), showing the highest response in fine‐textured soils (MD = 36.54 nodules plant^−1^), compared to medium‐textured soils (MD = 15.31 nodules plant^−1^). By contrast, soil pH did not show a significant effect on NN response to rhizobial inoculation (*P* > 0.05). However, neutral conditions favor greater NN in inoculated plants (MD = 39.29 nodules plant^−1^) than acidic (MD = 33.85 nodules plant^−1^) and alkaline (MD = 19.07 nodules plant^−1^) conditions. Similarly, EC had no significant effect on chickpea NN response to rhizobial inoculation (*P* > 0.05), with similar mean differences in non‐saline (MD = 26 nodules plant^−1^) and low‐saline soils (MD = 28.7 nodules plant^−1^). Otherwise, lower phosphorus (P_2_O_5_) availability significantly enhanced nodulation following inoculation (*P* < 0.001), as evidenced by greater NN response in low‐P_2_O_5_ soils (MD = 30.45 nodules plant^−1^) than in medium‐P_2_O_5_ soils (MD = 15.75 nodules plant^−1^).

**Figure 2:**
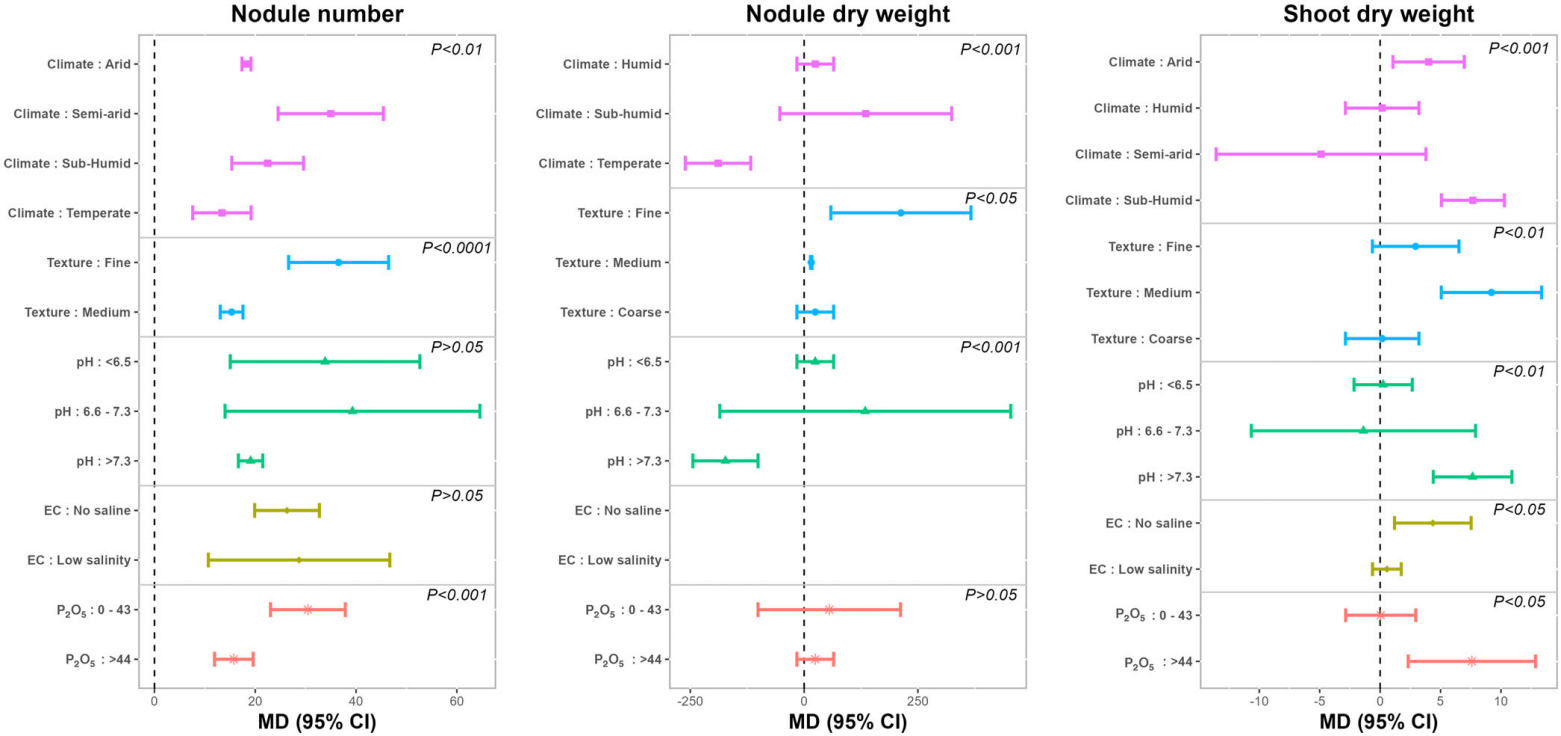
Mean difference (MD, *x*‐axis) of chickpea nodule number and nodule and shoot dry weight between observations conducted in inoculated and non‐inoculated conditions for different covariates (climate, texture, pH, electrical conductivity (EC), and available phosphorus (P_2_O_5_)). The error bars represent 95% CI confidence intervals. The dashed line corresponds to the no‐effect line (MD = 0). The significance of the *P*‐value of the overall effect is located at the top right of each covariate, with *P* > 0.05, not significant; *P* < 0.05, significant; *P* < 0.001, highly significant; *P* < 0.0001, very highly significant.

Under different climates, rhizobial inoculation significantly influenced NDW relative to non‐inoculated controls, particularly under optimal weather conditions (*P* < 0.001) (Fig. 2). The beneficial effect of chickpea inoculation on NDW was greater in a sub‐humid climate (MD = 135.44 mg plant^−1^) and medium in a humid climate (MD = 24.56 mg plant^−1^). However, a substantial negative response was observed under a temperate climate (MD = −89.1 mg plant^−1^). Otherwise, soil texture demonstrated a significant effect on NDW response to rhizobial inoculation (*P* < 0.05), especially under fine‐textured soils, indicating a greater response (MD = 212.63 mg plant^−1^) compared to coarse (MD = 24.56 mg plant^−1^) and medium‐textured soils (15.63 mg plant^−1^). Otherwise, soil pH strongly influenced the impact of rhizobial inoculation on NDW (*P* < 0.001). A maximum beneficial effect was observed in neutral soils (MD = 134.38 mg plant^−1^), a markedly lower response in acidic soils (MD = 24.56 mg plant^−1^), and a pronounced adverse response in alkali soils (MD = −72.81 mg plant^−1^). Concerning soil phosphorus content, unlike NN, SDW response did not show a significant difference following rhizobial inoculation (*P* > 0.05). However, a twofold response in low soil P_2_O_5_ (MD = 55.26 mg plant^−1^) relative to medium‐rich P_2_O_5_ soils (MD = 24.56 mg plant^−1^) was observed.

Rhizobial inoculation significantly affected SDW across bioclimate zones (*P* < 0.001) (Fig. 2). The highest response occurred in sub‐humid climate (MD = 7.67 g plant^−1^), followed by moderate response in arid climate (MD = 4.01 g plant^−1^), and negligible and negative responses in humid (0.17 g plant^−1^) and semi‐arid (MD = −4.89 g plant^−1^) climates, respectively. Regarding soil texture, a significant effect of response of SDW to rhizobial inoculation (*P* < 0.01) has been observed. The response was highest in medium‐textured soils (MD = 9.21 g plant^−1^), surpassing fine‐textured (MD = 2.94 g plant^−1^) and coarse‐textured soils (MD = 0.17 g plant^−1^). Similarly, soil pH significantly affected SDW response to rhizobial inoculation (*P* < 0.01), showing an opposite pattern to NDW. In alkali soils, rhizobial inoculation showed the highest increase in SDW (MD = 7.65 g plant^−1^) relative to non‐inoculated controls. However, acidic soils showed an insignificant response (MD = 0.25 g plant^−1^), while neutral soils exhibited a negative response (MD = −1.38 g plant^−1^). Otherwise, soil salinity significantly affected SDW response to rhizobial inoculation (*P* < 0.05), with the highest increase in non‐saline soils (MD = 4.36 g plant^−1^) relative to low‐saline soils (MD = 0.56 g plant^−1^). With respect to soil phosphorus, a significant effect on SDW response to rhizobial inoculation was observed (*P* < 0.05), as evidenced by highest increase in medium‐rich P_2_O_5_ soils (MD = 7.59 g plant^−1^) and no response in low‐P_2_O_5_ soils (MD = 0.05 g plant^−1^).

#### Effect of rhizobial inoculation on chickpea and seed protein

Rhizobial inoculation overall increased chickpea GY and SP by 28.1% and 7.48%, respectively, compared to non‐inoculated controls.

Under Mediterranean conditions, the climate significantly influenced GY response to rhizobial inoculation (*P* < 0.0001) (Fig. 3), showing an increase by 906, 592.5, and 467.85 kg ha^−1^ in sub‐humid, arid, and temperate climates, respectively. However, the response was negative in the humid and semi‐arid climates, exacerbating a decrease by 121.85 and 96.86 kg ha^−1^, respectively, compared to non‐inoculated controls. Otherwise, GY responded differently to rhizobial inoculation according to soil texture (*P* < 0.0001), showing the greatest increase in medium‐textured soils (MD = 1517.02 kg ha^−1^). However, fine‐ and coarse‐textured soils indicated different negative responses (MD = −4.46 and −779.96 kg ha^−1^, respectively). Results showed that rhizobial inoculation benefits on GY exhibited a significantly larger increase (MD = 1054.78 kg ha^−1^) under alkaline conditions (*P* < 0.05) than acidic (MD = 324.5 kg ha^−1^) and neutral‐pH soils (MD = 167.72 kg ha^−1^). Conversely, soil salinity did not significantly impact GY response to rhizobial inoculation (*P* > 0.05). However, a greater increase in no‐saline soils (MD = 672.47 kg ha^−1^) relative to low‐saline soils (MD = 444.11 kg ha^−1^) has been observed. By contrast, soil phosphorus content significantly influenced rhizobial inoculation efficacy (*P* < 0.05), showing the highest increase in medium‐rich P_2_O_5_ soils (MD = 1527.6 kg ha^−1^) *versus* low‐rich soils (MD = 471.61 kg ha^−1^), consistent with SDW patterns.

**Figure 3:**
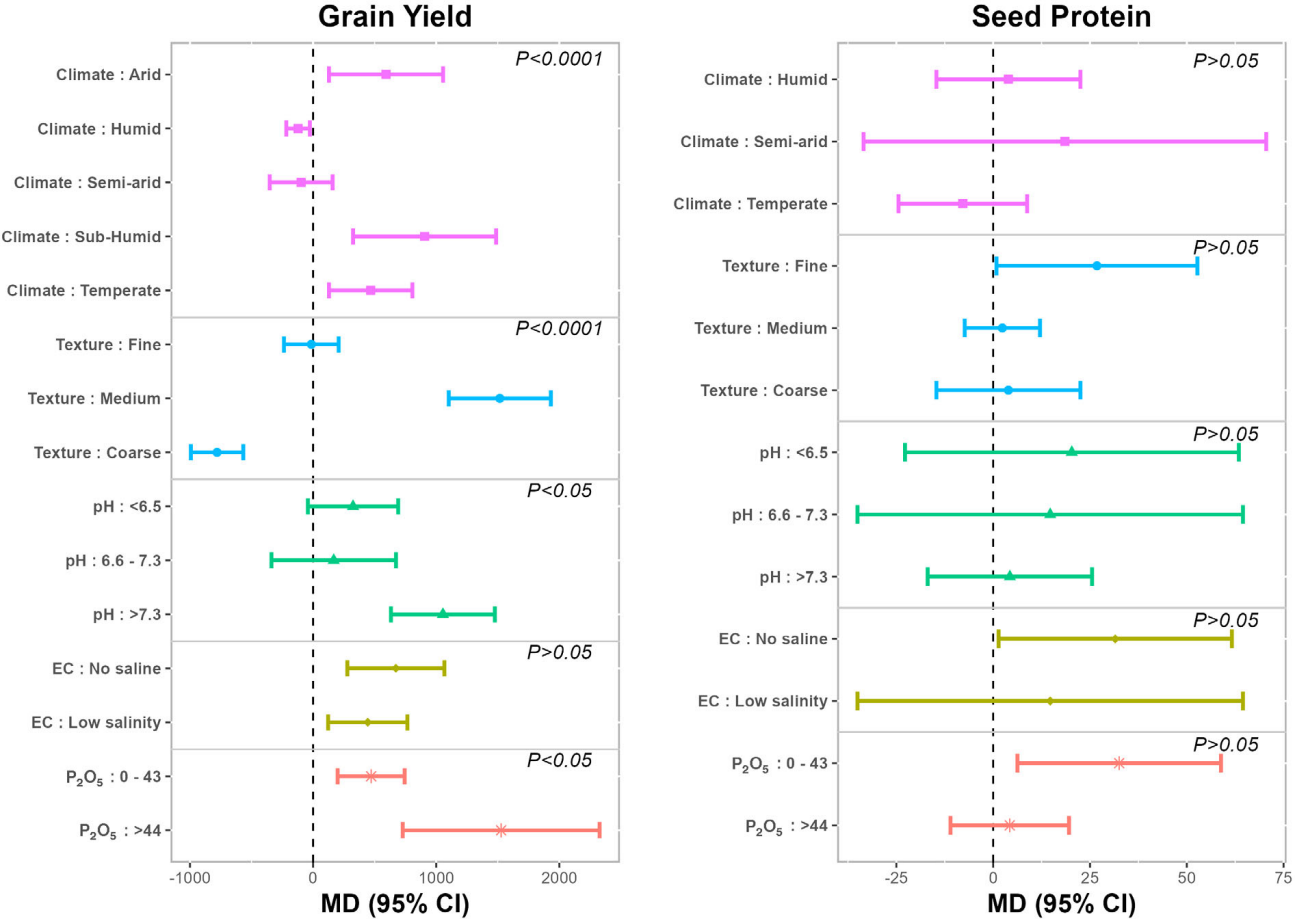
Mean difference (MD, *x*‐axis) of chickpea grain yield (kg ha^−1^) and seed protein (g kg^−1^) observations conducted in inoculated and non‐inoculated conditions for different covariates (climate, texture, pH, electrical conductivity (EC), and available phosphorus (P_2_O_5_)). The error bars represent the 95% CI confidence intervals. The dashed line corresponds to the no‐effect line (MD = 0). The significance of the *P*‐value of the overall effect is located at the top right of each covariate, with *P* > 0.05, not significant; *P* < 0.05, significant; *P* < 0.001, highly significant; *p* < 0.0001, very highly significant.

Seed protein was a unique parameter, showing no significant variation across different climates, soil textures, pH, EC, and P_2_O_5_ categories, in response to rhizobial inoculation (*P* < 0.05; Fig. 3). However, negligible positive responses of SP content were observed in semi‐arid (MD = 18.5 g kg^−1^) and humid climates (MD = 3.9 g kg^−1^) and a negative response in temperate climate (MD = −7.9 g kg^−1^). Concerning soil texture, the beneficial effect of rhizobial inoculation on SP content was negligible (MD = 9.7, 2.4, and 3.9 g kg^−1^ in fine‐, medium‐, and coarse‐textured soils, respectively). Conversely, when rhizobial inoculation was applied in acidic‐pH soils, SP content increased by 20.3 g kg^−1^. However, it increases by 14.8 g kg^−1^ in neutral‐pH soils and by 3.9 g kg^−1^ in alkali‐pH soils. As for soil salinity, inoculation response in SP content showed an increase by 32.5 g kg^−1^ in no‐saline soils and by 14.8 g kg^−1^ in low‐salinity soils. Otherwise, under low‐rich P_2_O_5_ soils conditions, rhizobial inoculation increased SP by 35 g kg^−1^, while it increased by 4.3 g kg^−1^ in medium‐rich P_2_O_5_ soils.

### Effect of inoculum type on chickpea nodulation, growth, and yield parameters

#### Inoculum type effect on chickpea NN and SDW

The forest plot in Fig. 4 showed significant differences in NN increase resulting from different inoculum types (*P* < 0.0001). The co‐inoculation using *Mesorhizobium* sp. in combination with *Enterobacter* sp. or *Bacillus* sp. resulted in the highest NN formed, with respective means of 86.67 and 79.24 nodules plant^−1^. This finding revealed a nearly doubled mean NN compared to single‐bacteria inoculation. Indeed, the inoculation using native *Mesorhizobium* sp./*Rhizobium* sp. strains, *Bacillus* sp., and *Enterobacter* sp. showed lower NN, with respective means of 34.67, 30.18, and 29.82 nodules plant^−1^.

**Figure 4:**
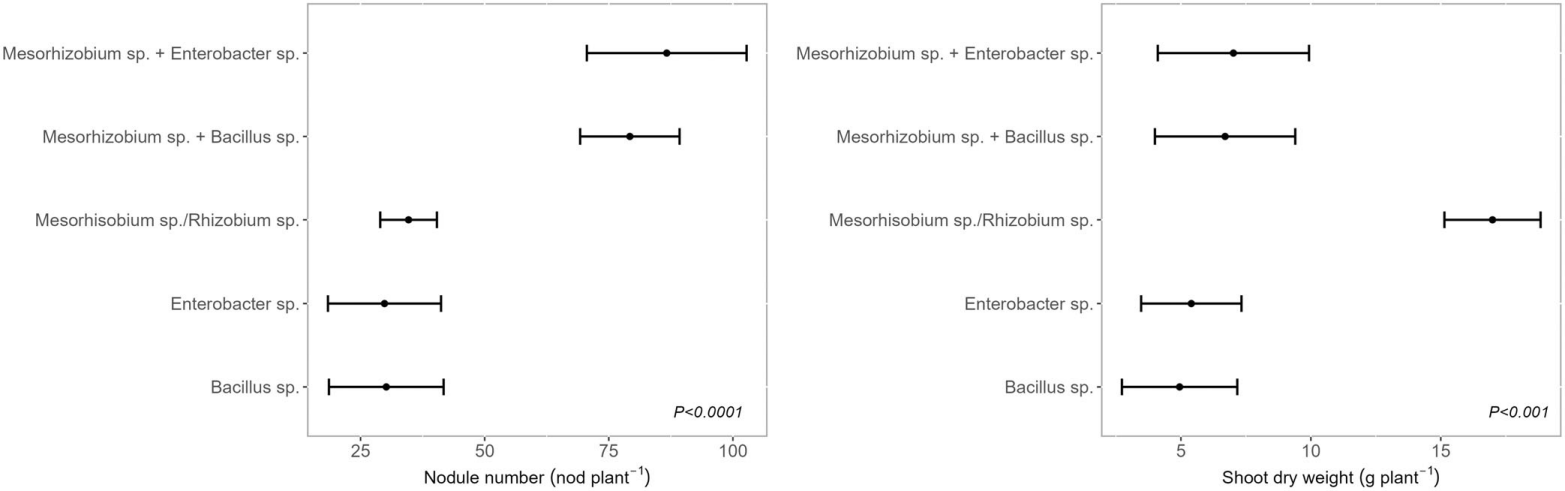
Nodule number (nodules plant^−1^) and shoot dry weight (g plant^−1^) of chickpea for different inoculum types (*Bacillus* sp., *Enterobacter* sp., *Mesorhizobium* sp./*Rhizobium* sp., *Mesorhizobium* sp. + *Bacillus* sp., and *Mesorhizobium* sp. + *Enterobacter* sp.). The error bars represent the 95% confidence intervals. The significance of the *P*‐value of the overall effect is located at the bottom right of each parameter, with *P* > 0.05, not significant; *P* < 0.05, significant; *P* < 0.001, highly significant; *P* < 0.0001, very highly significant.

The results showed that inoculant types induced significant variations in SDW (*P* < 0.0001), and *Mesorhizobium* sp./*Rhizobium* sp. inoculation displayed the highest mean (16.69 g plant^−1^; Fig. 4). However, combined inoculation using *Mesorhizobium* sp. + *Bacillus* sp. and *Mesorhizobium* sp. + *Enterobacter* sp. showed lower SDW, with respective means of 7.02 and 7.02 g plant^−1^. Additionally, *Enterobacter* sp. and *Bacillus* sp. alone led to very low SDW with respective means of 5.40 and 4.95 g plant^−1^.

#### Inoculum type effect on chickpea grain yield and seed protein content

The forest plots in Fig. 5 showed that all inoculum types significantly influenced chickpea grain yield (*P* = 0.0065). The dual inoculation using *Mesorhizobium* sp. + *Enterobacter* sp. and the single inoculation using *Mesorhizobium* sp./*Rhizobium* sp. achieved the highest GY mean values (2239.56 and 2365.35 kg ha^−1^, respectively), compared to single inoculation with *Bacillus* sp. or *Enterobacter* sp. The last achieved a mean GY of 1531.33 and 1605.9 kg ha^−1^, respectively. Furthermore, co‐inoculation using *Mesorhizobium* sp. + *Bacillus* sp. showed a moderate result, with a mean GY of 1940.61 kg ha^−1^.

**Figure 5:**
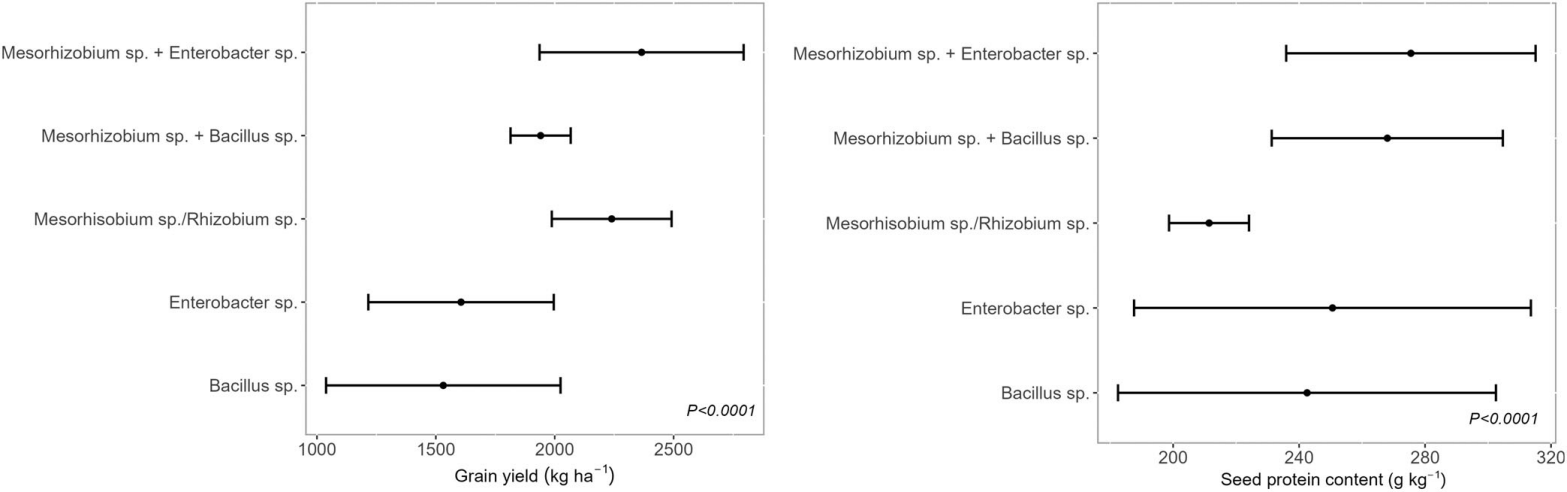
Grain yield (kg ha^−1^) and seed protein content (g kg^−1^) of chickpea for different inoculum types (*Bacillus* sp., *Enterobacter* sp., *Mesorhizobium* sp./*Rhizobium* sp., *Mesorhizobium* sp. + *Bacillus* sp., and *Mesorhizobium* sp. + *Enterobacter* sp.). The error bars represent the 95% confidence intervals. The significance of the *P*‐value of the overall effect is located at the bottom right of each parameter, with *P* > 0.05, not significant; *P* < 0.05, significant; *P* < 0.001, highly significant; *P* < 0.0001; very highly significant.

Similarly, our meta‐analysis revealed significant differences between inoculum types in their ability to improve chickpea seed protein content (*P* = 0.002) (Fig. 5). The combined bacterial inoculation significantly outperformed the single inoculation. The most substantial increase in SP content was observed when *Mesorhizobium* sp. was combined with either *Enterobacter* sp. (275.5 g kg^−1^) or *Bacillus* sp. (268 g kg^−1^). The single inoculation using native *Mesorhizobium* sp./*Rhizobium* sp. strains resulted in the lowest SP average (211.4 g kg^−1^), while inoculation with *Enterobacter* sp. and *Bacillus* sp. produced moderate SP averages (250.6 and 242.5 g kg^−1^, respectively).

## DISCUSSION

### Variation of chickpea NN, NDW, and SDW, and their response to rhizobial inoculation

This meta‐analysis highlights significant variations in chickpea responses to rhizobial inoculation across major studied covariates. It elucidates the high sensitivity of chickpea NN, NDW, and SDW to environmental conditions. Overall, chickpea NN response to introduced rhizobia was low, likely due to the widespread distribution of *Mesorhizobium ciceri* or *Mesorhizobium mediterraneum* across several Mediterranean countries or other abiotic factors.^14^ In our study, rhizobial inoculation under semi‐arid climates revealed the highest NN improvement with a negative effect on SDW, suggesting the high infectivity potential of introduced rhizobia, though limited by abiotic constraints. This indicates their limited adaptability to field conditions, resulting in low efficiency and poor establishment of symbiotic nitrogen fixation (SNF). However, arid climates exhibited moderate NN improvement with low impact on SDW, likely due to the low survival of introduced rhizobia. Temperate climates showed the weakest NN response alongside strongly negative effects on NDW and low response on SDW, probably due to low competitiveness and efficiency of introduced rhizobia and/or the presence of competitive specific rhizobia in the soil. Our study also revealed that humid climates resulted in negligible NDW improvement and no effect on SDW, possibly due to low competitiveness of inoculated strains or chickpea cultivar sensitivity to abiotic and biotic factors. Low and negative effects of rhizobial inoculation on chickpea nodulation and growth have been previously demonstrated in several studies.^25^, ^70^, ^71^, ^72^ Indeed, when rhizobia are introduced into the soil, a competition from the native microbial population may occur, affecting their biological functions.^23^ Conversely, inoculation responses for NDW and SDW were high under sub‐humid conditions. This reflects more favorable climatic conditions for SNF performance, including adequate water availability and temperature that enhanced rhizobia survival, their infectivity potential, nodulation, SNF and, in consequence, plant growth.^72^ In the Mediterranean region, drought and high temperatures are negatively affecting rainfed spring‐sown chickpeas by inhibiting rhizobia activity, nodule initiation, nitrogen‐fixing symbiosis and photosynthetic processes, and reducing dry matter yields.^73^, ^74^, ^75^, ^76^ Furthermore, inadequate soil moisture and temperature conditions disrupt rhizobial activity and nodule initiation, alter critical rhizospheric interactions, and significantly reduce the survival and infectivity of symbiotic bacteria.^77^, ^78^

Furthermore, under Mediterranean climate conditions, the response of NN and NDW to rhizobial inoculation was low in medium‐ and coarse‐textured soils (sandy clay loam and silty loam), with a greater effect in fine‐textured soils (clay and clay loam). These findings confirm the significant effect of soil matric potential and texture on chickpea rhizobia survival and activity, which is reported to more favored in fine‐textured soils than in coarse‐textured soils. This is due to the protective role of clay colloids against drought, which attract rhizobia through their positive charge.^79^ Moreover, in fine‐textured soils, greater water and substrate diffusion allows for better rhizobia growth and colonization by chickpea roots.^81^ Similar results have been reported by Gan *et al*.,^80^ who found that chickpea cultivation in clay soils under a semi‐arid climate increased nodule number by 37% compared to silty loam soils. Nodulation response increase under fine‐textured soils may also be caused by lower competitiveness of native rhizobia and/or the highest persistence and efficiency of introduced rhizobia. For instance, a study on bean inoculation with *Rhizobium elti* in different Egyptian soils showed increased NN and nodule biomass in clay soil compared with silty loam soil.^82^ Furthermore, SDW showed no response to rhizobial inoculation in coarse soils, low increase in fine‐textured soils, and the highest response in medium‐textured soils. These results corroborate those of Vargas‐Blandino and Cárdenas‐Travieso, reporting that chickpeas grow better in the moderately fine silty‐clay soils than in fine‐textured soils owing to lower clay content.^83^ In another study, inoculated legumes in sandy and clay soils showed lower nodule and shoot biomass response than in medium‐textured soils, owing to optimal soil moisture and higher rhizobial population activity.^25^, ^53^, ^56^ Additionally, chickpeas are particularly sensitive to waterlogging in clayey soils, leading to poorer root aeration, restricted rhizobial population size, limited nodulation, and decreased root growth and shoot biomass.^25^, ^84^

Soil pH showed no significant effect on NN response to rhizobial inoculation, indicating that pH may not be a determining factor for chickpea rhizobia persistence and nodulation stages. Indeed, *Mesorhizobium* strains commonly have a wide tolerance to pH ranges. The majority of the strains are tolerant to neutral and alkali pH (6.5–9.8), while a small number are tolerant to acid pH (4.5–5.5).^85^, ^86^, ^87^ It is reported that alkali‐tolerant *Mesorhizobium* strains can maintain intracellular pH through several mechanisms, including decreased membrane permeability, internal buffering, proton extrusion/uptake, amelioration of external pH, and prevention of metal ion toxicity.^85^ However, distinct responses to inoculation have been observed. Positive impacts on NN were observed only under neutral and acidic soil pH. Positive impacts on NDW under neutral pH, and negative impacts to negligible responses under alkali and acidic pH, were observed. Conversely, the highest positive impact on SDW under alkali pH and low impact to negative responses under acidic and neutral soils were observed, suggesting the good performance of chickpea SNF under alkaline pH. These findings are in line with previous studies demonstrating successful chickpea cultivation in alkaline soils^88^ and the deleterious effect of soil acidity for *Mesorhizobium* strains.^23^ Under acidic soil conditions, both rhizobia and chickpea roots are subject to nutrient deficiencies, particularly phosphorus, due to the binding of P to iron (Fe^3+^) and aluminum (Al^3+^), forming insoluble precipitates.^89^, ^90^ Moreover, nodulation steps, including attachment, root hair curling, and formation of infection threads, are very sensitive to soil acidity conditions, leading to reduced nodulation.^91^ Indeed, Al or Mn phytotoxicity may reduce rhizobia survival and persistence, disrupt host–rhizobia signaling, and affect root colonization, thereby reducing nodule formation and subsequent establishment of a viable SNF and plant growth.^84^, ^92^, ^93^, ^94^ These findings imply that, even with the use of acid‐tolerant rhizobia strains, symbiotic performance can be improved; however, acid‐tolerant chickpea cultivars should also be used to improve nodulation and plant growth.^92^

Regarding soil salinity, it had no significant effect on the global variation of chickpea NN, as well as its response to rhizobial inoculation, with close positive responses in non‐saline and low‐saline soils. However, SDW showed a positive response in non‐saline soils, but no response in low‐saline soils. These findings are due to the ability of some *Mesorhizobium* strains to survive under high‐salinity conditions, being more tolerant to salt stress than their chickpea host plants, which is considered as a salt‐sensitive legume.^74^, ^95^ Chickpea inoculation with *Mesorhizobium* strains possessing ACC deaminase activity can improve nodulation and plant growth under salinity conditions by reducing ethylene accumulation in plant cells.^21^, ^96^ However, salt‐stressed chickpeas and the nodule initiation process, which are both more sensitive to oxidative stress than rhizobia, can lead to decreased root and plant growth, weakened photosynthesis abilities, and a failure in the root infection process, resulting in reduced NN and biomass.^84^, ^97^ These events, driven mainly by oxidative damage from reactive oxygen species, compromise nitrogenase and leghemoglobin activity and thereby protein concentration in nodules, affecting oxygen transport to nodules, nodule efficiency, and symbiotic nitrogen fixation.^74^ Such a problem can be solved by using salt‐tolerant rhizobia and chickpea genotypes, which may display higher nodulation, growth and nitrogen fixation performance under salt stress owing to increased enzymatic antioxidant defense.^36^, ^93^, ^98^

With regard to phosphorus availability, the significantly higher NN response to rhizobial inoculation in environments with low P_2_O_5_ levels (<44 mg kg^−1^) can be attributed to the low competitiveness of native rhizobia, inhibited by P deficiency, and the high infectivity potential of introduced *Mesorhizobium* strains. However, the lack of SDW response to rhizobial inoculation in low‐P_2_O_5_ soils suggests the use of unadapted strains to soil P deficiency, which is crucial for SNF process and subsequent plant growth.^99^ This problem is common in calcareous Mediterranean soils, covering about half of the cultivated Mediterranean area, where P deficiency often results to stunted growth and poorly developed root systems.^100^, ^101^ Our findings are concordant with several studies indicating that P deficiency is a key constraint to chickpea nodulation and SNF. This is because chickpeas have high P demands, and nodules act as strong sinks for this nutrient, which is crucial for their development and the energy needed for SNF.^25^, ^36^, ^97^ The prevalence of ineffective rhizobia is a major constraint for farmers in low‐fertility Mediterranean soils, which restrict productivity.^102^ For instance, in Algerian soils, chickpea showed no nodules in a soil containing 14.11 mg kg^−1^ P_2_O_5_, while nodule biomass reached 380 mg in soils containing 29 mg kg^−1^P_2_O_5_. These results were explained by the absence of *Mesorhizobium* strains in P deficiency, along with the strong positive correlation between rhizobia population activity and nodulation with P availability.^25^ The same result was confirmed for native *Rhizobium leguminosarum* strains in calcareous Tunisian soils in relation to N and P deficiency.^35^ In this case, phosphorus fertilization or rhizobial inoculation using highly competitive, nitrogen‐fixing, and phosphorus‐solubilizing bacteria is crucial.^16^, ^35^

### Variation of chickpea grain yield and seed protein and their response to rhizobial inoculation

In the current study, the average chickpea grain yield for the studied sites under conventional rainfed Mediterranean conditions was very low, reaching 1821.08 ± 1038.57 kg ha^−1^; this is higher than global chickpea production (averaging 1000 kg ha^−1^) but far from chickpea potential (6000 kg ha^−1^).^10^, ^103^ This may be due to environmental variability, mainly climate changes and its consequences on pedological properties, as demonstrated in the previous sections. One study previously elucidated this challenge and found that, in two successive years in Spain, chickpea yield could reach 632 and 2190 kg ha^−1^ at the same site, and showed a strong positive correlation between yield and seasonal precipitation (*r* ^2^ = 0.74).^75^

Yield gap studies on conventional and rainfed chickpea crops revealed that yield variability can be the result of crop management practices, mainly variety adaptation, sowing method and rate, weed and pest management, and seed inoculation with *Rhizobium*.^104^ Besides, chickpea grain yield has been previously demonstrated to be strongly correlated with shoot biomass (*r* ^2^ = 0.9).^105^, ^106^ Thus, the highest positive response of GY to rhizobial inoculation specifically under sub‐humid climates (+906 kg ha^−1^) is probably related to the combined positive effect of climate and rhizobial inoculation on NDW and SDW. Otherwise, the lower efficacy of rhizobial inoculation under arid and temperate climates, as well as the negative response under humid and semi‐arid climates, could be attributed to reduced nodulation and plant biomass. This is in line with recent research reporting that low nodulation in Mediterranean areas is consistently considered as the key limiting factor of chickpea productivity.^25^ In our study, low‐yielding responses have been observed under contrasting Mediterranean climates. Irrespective of nodulation and plant biomass, during the reproductive phase, both cold conditions and drought may cause serious plant physiological changes and yield losses.^73^ For instance, low temperatures of <15 °C – prevalent in humid and temperate zones – significantly affect flower and pollen development and function, resulting in up to 50% yield losses.^84^, ^107^ Conversely, during flowering and grain filling, high temperature (35/18 °C, day/night) and subsequent soil water evaporation – frequent in Mediterranean semi‐arid regions – affects pod setting and reduces seed size and grain yields.^31^, ^75^, ^107^ It is reported that chickpea GY could decrease by 53–301 kg ha^−1^ per degree increase in temperature, with the absence of set pods when temperatures exceed 35 °C.^108^ The impaired sucrose metabolism and transport to developing pollen grains are the main causes of reduced pollen viability and poor pod setting, which may lead to 30–100% yield loss.^84^, ^107^

Chickpea GY response to rhizobial inoculation was significantly higher in medium‐textured soils, likely due to the highest SDW positive response owing to better plant water and nutritional status and a favorable environment to rhizobia and root growth.^79^ Otherwise, in fine‐ and coarse‐textured soils, where GY showed negative responses, plants could encounter physical constrains like limited porosity and, low moisture, compaction, clay dispersion, sodicity, and restricted root development. Additionally, chemical constraints such as salinity, alkalinity, calcareous soils, nutrient deficiencies, and ion toxicities, may create a hostile environment that limits chickpea growth and yield.^101^, ^109^ Already, it is reported that chickpea can adapt to a wide range of soil types; however, best yields are observed in fertile well‐drained soils.^84^, ^110^

Furthermore, rhizobial inoculation showed the highest positive response of GY in alkaline soils, with increases estimated at 3.25‐fold in acidic soils and 6.3‐fold in neutral soils. These findings suggest that inoculated *Mesorhizobium* strains are alkaline‐pH‐adapted, capable of maintaining nitrogenase activity and efficient nitrogen fixation, critical for plant growth, resulting in enhanced GY. In fact, in alkaline soils of arid and semi‐arid Mediterranean regions, low nutrient availability may decrease chickpea productivity.^111^ However, alkali‐tolerant *Mesorhizobium* strains can help plant nutrient acquisition and boost plant growth and yields by enhancing photosynthesis and leaf area.^85^

Although salinity did not show a significant difference in GY response to rhizobial inoculation in our study, a slight positive effect was observed in non‐saline soils, with no response in low‐saline soils, suggesting the use of both salt‐sensitive varieties and inoculated *Mesorhizobium* strains. Research has reported that even low salinity levels may lead to a 10% reduction in chickpea grain yields.^22^, ^109^ Under Mediterranean conditions, it has been found that chickpea response to salinity is cultivar‐dependent, altering both growth and yield components, including seed weight, branch number, filled pods, total pod count, and seed yield.^112^ Moreover, the *Mesorhizobium* has been cited among the most salt‐sensitive genus after *Bradyrhizobium*. However, they could have different tolerances to soil salinity, indicating their evolutionary adaptations to hostile environments.^22^, ^113^

Furthermore, chickpea GY response to rhizobial inoculation was significantly higher in medium‐rich P_2_O_5_ soils, compared to low‐rich soils, suggesting that the efficacy of rhizobial inoculation is soil P‐availability‐dependent. These results highlight the important role of chickpea P nutrition in achieving high yields, unveiling the restricted use of chickpea nutrient management strategies – mainly the inoculation with efficient P‐solubilizing *Mesorhizobium* strains (PSM).^111^ For instance, in phosphorus‐poor soils (27.6 mg kg^−1^ P_2_O_5_) in Turkey, fertilization with phosphorus at a rate of 80 kg ha^−1^ in combination with rhizobia increased chickpea grain yield by 22% compared to inoculation alone and by 25% compared to control. This suggests the use of non‐adapted *Mesorhizobium* strains in Mediterranean field conditions with P deficiency.^114^ Indeed, in alkaline calcareous soils – widely distributed in Mediterranean areas – several nutrients, including N, P, Fe, Zn, Cu, and B, are usually deficient, making the use of phosphorus‐solubilizing *Mesorhizobium* strains crucial for effective symbiotic nitrogen fixation and potential yields. Indeed, PSM enhance solubilization of fixed phosphorus through various mechanisms, such as rhizosphere acidification, organic acids production, protons release, mineralization through alkaline and acid phosphatases, and chelation.^115^

Even though edapho‐climatic factors showed no significant variation in chickpea seed protein (SP) content response to rhizobial inoculation, a low positive effect was observed in a semi‐arid climate, with negligible and negative responses in humid and temperate climates, respectively. This aligns with recent research reporting that water availability did not affect chickpea SP content; however, drought conditions can increase protein and nutrient (Fe, Zn, P, and Ca) levels, due to various physiological processes, such as increased antioxidant enzymes levels, heat shock proteins, and osmo‐protectants like proline and betaine.^116^ Moreover, assessment of the effect of *Rhizobium*, PGPR, and P fertilization, and different combinations on chickpea SP content, showed no significant effect of all treatments compared to the control.^117^ Otherwise, we noticed that rhizobial inoculation had the highest positive effect on SP content in fine‐textured, acidic, non‐saline, and low‐P_2_O_5_ soils. This response displayed a visually apparent inverse pattern of SDW and GY. Our results suggest that chickpea SP content negatively correlates with GY, indicating a compromise between protein content, seed size, and grain yield, necessitating the focus on the selection of moderate yields and high‐protein genotypes in future breeding programs.^29^

### Effect of inoculum type on chickpea NN and SDW

Our meta‐analysis revealed that, under Mediterranean conditions, inoculum type significantly influenced chickpea NN and SDW. Dual inoculation using *Mesorhizobium* sp./*Rhizobium* sp. in combination with PGPB outperformed the single inoculation with *Mesorhizobium* sp./*Rhizobium* sp., *Bacillus* sp., or *Enterobacter* sp. in nodule formation. This emphasizes the synergetic effect of rhizobia with PGPB in enhancing nodulation under challenging Mediterranean conditions. However, the single inoculation using *Mesorhizobium* sp./*Rhizobium* sp. achieved the highest SDW. This highlights the essential contribution of highly efficient nitrogen‐fixing rhizobia and effective symbiotic nitrogen fixation in promoting chickpea growth. Our results corroborate findings of Aulakh *et al*.,^118^ who found that mixed inoculation of chickpea using *Mesorhizobium ciceri* and *Enterobacter cloacae* increased nodule biomass by 18% and SDW by 15.1% compared to *Mesorhizobium ciceri* and *Bacillus subtilis* consortium. This is because microbial consortia are composed of unique mixtures of bacteria which respond and adapt to ambient and soil‐related environmental stresses owing to their synergetic interactions with soil microbial communities.^23^

In the present study, the low performance of PGPB single inoculation has been confirmed by other research, indicating that chickpea inoculation with *Bacillus* species decreased shoot biomass by 22%, leaf number, and branches, compared to control. This suggests the presence of free‐living bacteria in the soil competing chickpea plants and inoculated strains for nutrients and soil carbon resources.^119^ Furthermore, *Bacillus* species and *Enterobacter agglomerans* are widely used in pathogen bio‐control.^120^ Additionally, due to their phytohormone release, single inoculation of *Bacillus* has been shown to be effective in enhancing nodulation, growth, photosynthesis, leghemoglobin, grain yield, and seed protein of chickpea grown in metal‐contaminated soils.^121^ On the other hand, *Enterobacter* sp. has been found to promote chickpea growth through enhancing root growth and plant biomass by maximizing nutrient uptake, mainly N, P, K, Ca, and Na, and the synthesis of phytohormones and enzymes solubilizing soil organic nutrients.^120^

As for *Mesorhizobium* sp., it has been demonstrated to promote chickpea growth through N_2_ fixation, phosphorus solubilization, siderophore, HCN, cytokinin and indoleacetic acid (IAA) production, phytoremediation, and plant tolerance to environmental stresses, including low/high temperature, salinity, acid/alkaline pH, etc.^19^, ^88^, ^96^, ^120^, ^122^, ^123^ Research indicates that multi‐strain inoculants, possessing a multi‐functional activity and broader adaptation to environmental stresses, have greater potential to benefit chickpea nodulation, plant growth, and health promotion. They operate through several mechanisms, rather than individual microorganisms, acting in a single way.^15^, ^23^, ^120^, ^124^, ^125^ In a recent study, chickpea inoculation with *Mesorhizobium cicer* + *Pseudomonas triata* consortia achieved the highest significant N, P, and K plant nutrient content and uptake. It also produced the greatest straw yield compared to *Rhizobium* spp. + *Bacillus megaterium*, *Rhizobium* spp. + *Thiobacillus thiooxidans*, and control. This was due to a higher potential for organic acid secretion, which increased nutrient availability and soil organic carbon.^126^ In other research, chickpea dual inoculation with *Mesorhizobium* and *Pseudomonas* sp. showed significant improvement of seed germination, plant height, nodule occupancy, nodule number, nodule dry weight, leghemoglobin content, and plant N and P content, over single *Mesorhizobium* sp. and *Pseudomonas* sp. inoculation. This effect was attributed to PGPB releasing plant growth regulators, such as phytohormones (IAA), which regulate root and shoot growth and support rhizobia survival. Additionally, siderophore production facilitated iron uptake, needed in leghemoglobin and nitrogenase biosynthesis. Moreover, increased flavonoid levels enhance nodulation. Finally, organic acids and proton release by *Rhizobium* during symbiotic N_2_ fixation reduces soil pH and increases P and nutrient availability.^124^ Similarly, Egamberdieva *et al*. and Sivaramaiah *et al*. demonstrated increases in NN, shoot and root length, and SDW by ratios of 1.62–1.74, as well as N and P uptake and symbiotic effectiveness of *Mesorhizobium* sp. when co‐inoculated with *Bacillus* sp. strains, compared to single *Mesorhizobium* inoculation.^21^, ^127^ Confirming our results, Mirza *et al*. found a maximum increase in RDW and SDW, plant height and NN in chickpeas dual inoculated with *Rhizobium* and *Enterobacter* over *Rhizobium* alone and control.^128^

### Effect of inoculum type on chickpea grain yield and seed protein content

This meta‐analysis demonstrates that the type of inoculant significantly affected chickpea GY and SP, with dual inoculation using *Mesorhizobium* sp./*Rhizobium* sp. + *Enterobacter* consortia achieving the highest results. Conversely, single inoculation with *Mesorhizobium* sp./*Rhizobium* sp. resulted in high GY and the lowest SP content. Moreover, single inoculation with *Bacillus* sp. or *Enterobacter* sp. resulted in the lowest GY and moderate SP content. These findings demonstrate the beneficial effect of consortia inoculants on modulating key plant physiological processes, leading to both enhanced yield and seed quality in chickpea. Our findings are consistent with other research indicating that triple inoculation of chickpea with *Rhizobium*, *Bacillus subtilis*, and *Bacillus megaterium* resulted in better nodulation and yield than *Rhizobium* alone, and similar results to chemical fertilization. It also showed an increased GY by 18% for *Rhizobium* alone, by 30.5% for triple strain consortium, and by 33.1% for recommended NP fertilization, over the control.^16^ Similarly, co‐inoculation of *Mesorhizobium* with *Bacillus megaterium* has been shown to significantly increase pod and seed yield of chickpea.^21^ Furthermore, chickpea inoculation with *Bacillus* alone showed an increase in SP content, from 224 to 249 g kg^−1^ under metal‐contaminated soil conditions.^121^ A recent study under arid and water‐stressed field conditions demonstrated that chickpea consortia inoculation with *Mesorhizobium cicer* + *Enterobacter cloacae* + *Providencia vermicola* increased pod number from 17.7 to 37.3 pods plant^−1^ and grain yield from 335.8 to 1179.25 kg ha^−1^, over non‐inoculated control. Conversely, a consortium of *Mesorhizobium cicer* + *Bacillus subtilis* + *Bacillus mojavensis* exhibited slightly lower performance under the same conditions. These results were evidenced by the higher plant‐growth‐promoting potential of *Enterobacter cloacae*, showing increased production of IAA, exopolysaccharides, and ACC deaminase, and the highest P‐solubilizing activity, which helped mitigate drought stress.^118^ Overall, this meta‐analysis suggests that co‐inoculation strategies, particularly involving diazotrophic and plant‐growth‐promoting rhizobacteria (PGPR), can enhance chickpea performance more effectively than single strains, supporting their use as climate‐resilient biofertilizers in Mediterranean cropping systems.

## CONCLUSIONS

By assessing the efficacy of rhizobial inoculation on nodulation, biomass accumulation, grain yield, and seed protein content of conventional chickpeas grown in rainfed Mediterranean areas, the results of this meta‐analysis indicate that – due to the adaptative response of inoculants and that of the chickpea crop to environmental factors – the potential proposed benefits are limited to specific edapho‐climatic conditions. In this study, we identified the climate zone, soil texture and pH, soil P content, and salinity as determining factors for rhizobial inoculation efficiency in Mediterranean chickpeas. However, more experiments on selecting site‐specific or tailored *Mesorhizobium* strains are needed, with the exploration of PGPR consortia such as *Mesorhizobium* sp. + *Enterobacter* sp. for effective symbiotic nitrogen fixation and enhanced chickpea yields and seed proteins simultaneously. Further research is needed to elucidate the effect of other factors on chickpea rhizobial inoculation efficacy. They particularly concern the genus of introduced rhizobia, its competition with the indigenous microbiome, the inoculation methods, and various crop management practices, including chemical fertilization (N and P), pest and weed control, chickpea genotype, and sowing rate, method, and date. In conclusion, this meta‐analysis provides critical insights into chickpea–rhizobia interactions across diverse environments, helping guide future sustainable agricultural strategies. The findings highlight key pedo‐climatic factors to consider for improving inoculant performance, contributing to food security and environmental sustainability in the Mediterranean region and similar agroecosystems. Continued research in this area will be required to harness the full potential of symbiotic nitrogen fixation for resilient chickpea production under changing climatic conditions.

## CONFLICT OF INTEREST

The authors declare that they have no conflicts of interest.

## Data Availability

The data that support the findings of this study are available from the corresponding author upon reasonable request.
